# The Prognostic Value of Coronary Arteries Calcium Scoring in a Primary Health Care Setting in Riyadh, Saudi Arabia: A Retrospective Cohort Study

**DOI:** 10.7759/cureus.25623

**Published:** 2022-06-03

**Authors:** Nora Alalem, Abdullah Alkhenizan, Loay Basudan, Fareeha Amin, Suad Alsoghayer

**Affiliations:** 1 Family Medicine, King Faisal Specialist Hospital and Research Centre, Riyadh, SAU; 2 Family Medicine and Polyclinics, King Faisal Specialist Hospital and Research Centre, Riyadh, SAU

**Keywords:** ascvd risk, cardiac risk, qrisk, coronary artery calcium score, coronary risk

## Abstract

Background/Purpose: Coronary Artery Calcium Scoring (CACS) by CT, the American Atherosclerotic Cardiovascular Disease (ASCVD) Score, and the British Cardiovascular Risk (QRISK2) score are the most frequently used cardiovascular risk stratification scores to predict cardiac outcomes and aid in the decision of implementing preventative and/or interventional measures. The aim of this study is to assess CACS, ASCVD score, QRISK2 score, and their capacity to predict cardiovascular events among family medicine patients in King Faisal Specialist Hospital and Research Centre (KFSH&RC), Riyadh, Saudi Arabia.

Methodology: All medical records of patients (18 years and above) who had a CACS done in Family Medicine Clinics at KFSH&RC from January 2010 to March 2018 were reviewed, retrospectively. The study variables included demographics, comorbidities, CACS, ASCVD Score, QRISK2 score, and cardiovascular events.

Results: We included 218 patients. Our study population included: 77% men, a mean age of 51 years (SD±8), and a mean BMI of 29 kg/m2 (SD±5). CACS was significantly associated with coronary events (p-value < .05). There was significant association between high CACS (>400) and family history of cardiac disease (p-value = .006), prior cardiovascular events (p-value = .01) and advancing age (p-value < .001). High concordance was found between QRISK2 score and CACS (90.6%), and moderate concordance between ASCVD score and CACS (69.4%). Moderate concordance was found between ASCVD score and QRISK2 score (74.3%). The majority of the subjects (88%) fell into the low-risk group (CACS <100) with (63%) having a CACS of zero.

Conclusion: QRISK2 cardiac assessment tool provides better risk assessment and higher concordance with CACS. To improve cost-effectiveness and minimize unnecessary radiation exposure, QRISK2 scoring should be implemented for initial cardiovascular risk stratification prior to ordering the CACS imaging modality.

## Introduction

Coronary artery disease in the form of fatal myocardial infarction (MI) is the leading cause of death worldwide [1]. In Saudi Arabia, it is estimated that 20% die from MI, making it the number one cause of death from 2005 to 2016 [2]. At least 25% of patients experiencing nonfatal acute myocardial infarction had no previous symptoms [3]. Multiple risk stratification methods and scores have been researched and implemented in this field to better prevent fatal outcomes by early detection and management [3,4]. Such methods include the Framingham risk assessment score (FRS), the QRISK2 assessment score, the Atherosclerotic and Cardiovascular Disease (ASCVD) risk assessment score, and the Coronary Artery Calcium Score (CACS) [5].

The characterization of coronary artery calcification by computed tomography shows equivalence with the total coronary atherosclerosis load and the risk of cardiovascular events [6]. CACS is a relatively new method of screening. It implements computed tomography imaging of coronary arteries and detection of Calcium deposits in the arteries, measurement of plaque deposition, and level of occlusion, to calculate a score from zero to more than 400. The score corresponds to the level of occlusion and the risk percentage of patients to develop cardiac events in the future, known as the Agatston method, which is implemented in King Faisal Specialist Hospital and Research Centre (KFSH&RC) [7]. A score of zero corresponds to no plaque; 1-10 represents a small amount of plaque with a chance of heart disease that is less than 10%; 11-100 means plaque is present with mild heart disease and moderate risk of heart attack; 101-400 indicates a moderate amount of plaque that may be blocking an artery and a moderate-to-high chance of having a heart attack. Finally, a score of over 400 means a large plaque is present and there is a greater than 90% chance of complete blockage of one of the arteries and a high chance of having a heart attack in the future [8]. The CACS is also an independent predictor of the risk of major cardiovascular events, with demonstrated superiority over the Framingham risk score, C-reactive protein level, and carotid intima-media thickness [9-14].

The use of the CACS in asymptomatic subjects at intermediate risk, as determined by traditional clinical stratification methods such as the Framingham risk score, is considered appropriate/recommended with a good level of evidence by the II Guidelines of the Brazilian Society of Cardiology/Brazilian College of Radiology and Diagnostic Imaging and other international consensus statements [14-19]. The use of the CACS is not indicated in high-risk patients, because aggressive preventive measures would already be indicated in such patients [4].

Various studies have shown that asymptomatic patients with a CACS of zero have a low risk of cardiovascular events or all-cause mortality in the medium and long term. However, there are still no recommendations to limit the use of preventive measures, such as lipid-lowering medications, if the patient is classified as being at intermediate or high risk by the traditional scores [4,20,21]. Among low-risk groups, recent evidence has shown that a family history of premature coronary artery disease (CAD) (in a male first-degree relative < 55 years of age or female first-degree relative < 65 years of age) is an independent risk factor and is associated with increased atherosclerotic burden [4].

A previous study in Beijing, China, and Torrance, California, addressed the prognostic value of coronary CT angiography and Calcium score for major adverse cardiac events in the outpatient population. The study followed 4.425 patients and found that as the CACS increased, the probability of composite cardiac death, nonfatal MI, or coronary revascularization increased (CACS > 400) [22]. A meta-analysis of four studies done in 2004 yielded a summary adjusted relative risk (RR) of 2.1 for CACS from 1-100 and an RR ranging from 3-17 for higher CACS. They concluded that CACS is an independent predictor of coronary heart disease events [23].

In Saudi Arabia, a few studies have been conducted regarding this topic. Among them, a retrospective analysis of CACS was performed in King Faisal Specialist Hospital in 2015 that examined 918 asymptomatic women with CAD risk factors; the women all underwent screening by CACS. They were found to be in the 75th and 90th centile of CACS, placing them at a higher chance of developing a heart attack; this is significantly more than the rest of the world [24]. Another study in AlQassim used cross-sectional analysis to compare CACS as the gold standard to other cardiac risk stratification scores. It included Framingham, the American College of Cardiology/American Heart Association (ACC/AHA) Pooled Cohort Risk Equation, and the European Systemic Coronary Risk Evaluation Score. The ACC/AHA Pooled Cohort Risk Equation was found to be more sensitive than the others [25]. No study regarding this subject was conducted in primary care settings in Saudi Arabia. This study aimed to evaluate risk factors that predict a higher CACS among the Saudi population, average CACS, and the prognosis and outcome of patients with different CACS values. 

## Materials and methods

The study design used was a retrospective cohort chart review. It took place in the family medicine and polyclinic department in King Faisal Specialist Hospital and Research Center in Riyadh, Saudi Arabia. The whole population was included (218 subjects). Inclusion of all subjects above 18 years of age who had a CACS arranged by the department of family medicine and polyclinics from January 2010 to December 2018. The research electronic data capture (REDcap; https://projectredcap.org/) program was utilized for data collection, then extracted into the SPSS software, version 21.0 (IBM Corp., Armonk, NY).

Study variables

The following study variables were included: The Agatston Calcium CT score reported as a numerical value by King Faisal Specialist Hospital’s Radiology department in their reports; age, body mass index, prior existing comorbidities (diabetes, hypertension, dyslipidemia, atrial fibrillation, chronic kidney disease, rheumatoid arthritis), patients on treatment for these comorbidities, levels of LDL, total cholesterol, high-density lipoprotein (HDL), hemoglobin A1C (HbA1c), systolic and diastolic blood pressure, prior obtained Calcium CT score date (closest to the Calcium score up to 12 months prior); Known cardiovascular risk factors (current, ex, or nonsmoker status, prior cardiovascular disease history, family history of cardiovascular disease or premature cardiac death in a first-degree relative, defined as death from a heart attack before 65 years for women and 55 years for men); Prior cardiac imaging and their findings (normal or abnormal).

We calculated the Atherosclerotic Cardiovascular Disease risk estimator score and the QRISK2 score for each patient based on the previously mentioned variables. Documented cardiovascular events (stroke, acute coronary syndrome) after the Calcium CT score date were recorded.

The data was extracted into an SPSS sheet. Preliminary descriptive analysis was done to identify outliers and data that needed further cleaning. The ASCVD score was grouped into three categories based on the American college of cardiology and the American Heart Association (low risk <7.5%, moderately high risk 7.5-14.9%, and high risk =>15%). The QRISK2 score was divided into two groups based on previous studies identifying the cutoff of 20% (low risk <20% and high risk =>20%).

As for the Agatston score, it is grouped by the American Radiology Foundation as: negative score = 0, minimal risk 1-10, low risk 11-100, moderate risk 101-400, and high risk >400). Control of lipid profile, HbA1c, and blood pressure was grouped into normal or controlled and abnormal or non-controlled based on the ATP, ADA, and GNC8 guidelines respectively. A second preliminary descriptive analysis was done to proofread the cleaned dataset.

Data analysis

Descriptive analysis was done using the SPSS software, version 21.0. Continuous data were reported as the maximum value, minimum value, mean and standard deviation. Categorical variables were reported as number and frequency percentages. Association analysis was done using linear regression analysis (continuous Calcium CT score and continuous variables) as well as Logistic regression analysis (categorical Calcium CT score with 400 as the cutoff for high and low to moderate and continuous variables). Chi-Square between Calcium CT score as categorical and other categorical variables. P-values equal to or greater than 0.05 was declared significant. Concordance between the three cardiovascular risk stratification scores was determined by proportional relationships between (1) ASCVD and Calcium CT score, (2) QRISK2 and Calcium CT score, and (3) ASCVD score and QRISK2 score. They were viewed as the previously mentioned categories and the Calcium score cutoff for high and low to moderate was 400. The mean follow-up duration was calculated from the date of the Calcium score to the end of the data collection time (December 2018).

## Results

The baseline characteristics are shown in Table 1. The mean age of the patients was 51.3 ± 8 years, with 77.5% of them being men. Coronary artery events were present in 12 (5%).

**Table 1:** Population demographics and characteristics

Variable	Mean & Std. Deviation
Age	51.3 ± 8.3
Body Mass Index	29.4 ± 5.1
Hemoglobin A1C	6.05 ± 1.25
Low-density Lipoprotein	3.3 ± 0.94
High-density Lipoprotein	1.2 ± 0.35
Total Cholesterol	4.9 ± 1

Distribution of calcium scores

The majority of subjects had a negative score of zero (63%). Low, moderate, and intermediate-risk Calcium score categories were occupied by 26%, 7%, and 4% respectively.

Distribution of coronary events among different calcium scores

Higher Calcium scores were significantly associated with coronary events (p-value <0.05). Among study subjects who developed coronary events, 41% had high CACS and 42% had moderate, 17% were negative. The association with a higher Calcium score is shown in Tables 2-3. Advancing age and prior cardiac history were significantly associated with higher Calcium scores.

**Table 2:** Chi-square association between categorical variables and CACS as high and low categories CACS: Coronary Artery Calcium Score

Variable	Percentage %	p-value
Gender (male)	77.5	.052
Family History	11.5	.006
Prior Cardiac History	3.2	.010
Smoking	16.5	.492

**Table 3:** Linear regression association between continuous variables and CACS as a continuous number OR: Odds ratio; CACS: Coronary Artery Calcium Score

Variable	OR	95% CI	p-value
Age	3.9	1.6 –3.9	.000
Body mass index	1.4	-1.7 – 11.4	.146
Hemoglobin A1c	.53	-20.8 – 36.2	.594
Low-density Lipoprotein	-2.1	-60.8 – -2.6	.033
High-density Lipoprotein	-1.05	-121.1 – 40	.295
Total Cholesterol	-1.94	-53.7 – .388	.053

Control of subjects

Blood pressure, HbA1c, LDL cholesterol, and cholesterol levels were in the target range for age and gender as can be seen in Table 4.

**Table 4:** Population control of comorbidities

Variable	Percent %
Controlled Hemoglobin A1C	87.1
Controlled Blood Pressure	79.2
Controlled LDL	78.5
Controlled Total Cholesterol	88.3

Concordance among scores

**Figure 1:**
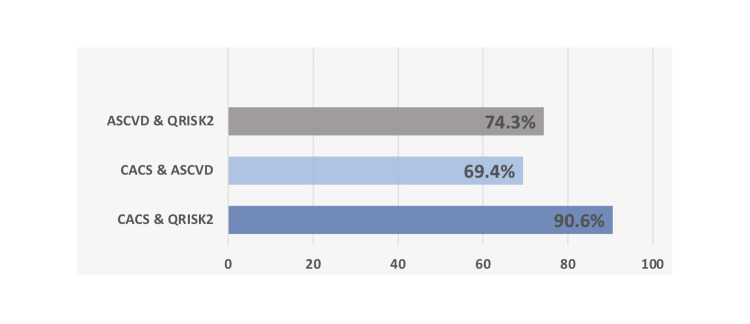
Concordance among ASCVD score, QRISK2 score and CACS ASCVD: American Atherosclerotic Cardiovascular Disease; Cardiovascular Risk Score: QRISK2; CACS: Coronary Artery Calcium Score

Although both QRISK2 and ASCVD scores had a good concordance with CACS, QRISK2 had a higher concordance with CACS over ASCVD with 90.6% over 69.4%. Between QRISK2 and ASCVD scores the concordance was 74.3%. Concordance among different scores and Calcium scores is presented in Figure 1.

## Discussion

In this study, we compared two cardiovascular disease risk stratification tools to each other and to the coronary Calcium CT score. The concordance between the two calculated scores was 74.3%, which was moderately high. When we compared the QRISK2 and the ASCVD scores in terms of concordance with CACS, both had good concordance, but QRISK2 showed better concordance at 90.6% over 69.4% of the ASCVD score.

The main associated risk factors were similar to what is found in the literature: age, male gender, and prior cardiac history. However, in our study, LDL was inversely related to coronary events; this contradicted the common link known to exist between LDL and heart disease. This can be explained through our previously mentioned results regarding the control of our subject’s comorbidities, including LDL levels. Among the prior studies worldwide, a study in the UK compared QRISK2, Framingham, and ASSessing cardiovascular risk using SIGN (ASSIGN) scores [26]. Overall, Framingham overestimated cardiovascular disease by 34%, ASSIGN by 36%, and QRISK2 by 0.4%; this made QRISK2 the most accurate, similar to our study. In the USA, a study compared the Framingham, adult treatment panel III (ATP III), Reynold’s, and ASCVD scores [27]. None of the previously mentioned showed accurate predictions for males or females. We can conclude from our study that the QRISK2 score seems to be more suited to predicting cardiovascular risk in Saudi populations prior to ordering Calcium CT scores.

Study limitations

The limitations of our study are the lack of proper documentation of personal cardiac history and/or family history of premature cardiac deaths, as well as missing lab values during or around the time CACS was done. Also, any positive coronary event outside the hospital that was not known to the authors was not taken into account. We recommend additional larger studies be performed in this field to better assess which cardiovascular risk stratification tool is best suited to the Saudi population.

## Conclusions

QRISK2 cardiac assessment tool provides better risk assessment and higher concordance with CACS. To improve cost-effectiveness and minimize unnecessary radiation exposure, QRISK2 scoring should be implemented for initial cardiovascular risk stratification prior to ordering CACS by CT. Better control of the cardiovascular system (CVS) risks is recommended throughout the Kingdom of Saudi Arabia's (KSA’s) primary care centers.
